# A Promising Antifungal and Antiamoebic Effect of Silver Nanorings, a Novel Type of AgNP

**DOI:** 10.3390/antibiotics11081054

**Published:** 2022-08-03

**Authors:** Sara González-Fernández, Victor Lozano-Iturbe, Mª Fe Menéndez, Helena Ordiales, Iván Fernández-Vega, Jesús Merayo, Fernando Vazquez, Luis M. Quirós, Carla Martín

**Affiliations:** 1 Department of Functional Biology, University of Oviedo, 33006 Oviedo, Spain; saragonzalezfernandez7@gmail.com (S.G.-F.); lozanoiturbevict.fuo@uniovi.es (V.L.-I.); helenaordiales@gmail.com (H.O.); fvazquez@uniovi.es (F.V.); 2 Instituto Universitario Fernández-Vega, University of Oviedo, Av. Drs Fernández Vega 34, 33012 Oviedo, Spain; fernandezvivan@uniovi.es (I.F.-V.); merayo@fio.as (J.M.); 3 Department of Photonics, ITMA Materials Technology, 33490 Avilés, Spain; marife.menendez@idonial.com; 4 Department of Pathology, Hospital Universitario Central de Asturias, 33011 Oviedo, Spain; 5 Instituto de Investigación Sanitaria del Principado de Asturias, Av. del Hospital Universitario, s/n, 33011 Oviedo, Spain; 6 Department of Surgery, University of Oviedo, 33006 Oviedo, Spain; 7 Department of Microbiology, Hospital Universitario Central de Asturias, 33011 Oviedo, Spain

**Keywords:** silver nanoparticles, silver nanorings, silver nanospheres, silver nanowires, antifungals, antiamoebics

## Abstract

Silver nanoparticles (AgNPs) play an important role in the medical field due to their potent antimicrobial activity. This, together with the constant emergence of resistance to antimicrobial drugs, means AgNPs are often investigated as an alternative to solve this problem. In this article, we analyzed the antifungal and antiamoebic effects of a recently described type of AgNP, silver nanorings (AgNRs), and compared them with other types of AgNPs. Tests of the activity of AgNPs against various fungal and amoebic species were carried out. In all cases, AgNPs showed a high biocidal effect, although with fungi this depended on the species involved. Antifungal activity was detected by the conditioning of culture media or water but this effect was not dependent on the release of Ag ions. On the other hand, the proliferation of *Acanthamoeba castellanii* trophozoites was reduced by silver nanorings (AgNRs) and silver nanowires (AgNWs), with AgNWs being capable of totally inhibiting the germination of *A. castellanii* cysts. AgNRs constitute a new type of AgNP with an antifungal and antiacanthamoebic activity. These results open the door to new and effective antimicrobial therapies as an alternative to the use of antifungals or antiamoebic drugs, thus avoiding the constant appearance of resistance and the difficulty of eradicating infections.

## 1. Introduction

In recent years, nanotechnology has been in continuous development and recent advances show that nanoparticles (NPs), specifically silver nanoparticles (AgNPs), are playing and will play an important role in the medical, biological and pharmaceutical fields. NPs are between 1–100 nm and the increase in the use of AgNPs compared with other metal NPs is due to their physical-chemical characteristics and the well-known toxic effect of silver, which gives them good antimicrobial capabilities [1,2,3]. Depending on how they are synthesized, their size, and their morphology, AgNPs have unique and different characteristics, which allows them to be applied in a wide range of functions such as biosensors and anticancer therapy, in bioimaging, wound healing, disease treatment and drug delivery, or as nutraceuticals [4,5]. To date, several studies described the potent antimicrobial activity of AgNPs against bacteria, considering them an effective alternative to dealing with the problem of bacterial multidrug resistance such as ampicillin-resistant *Escherichia coli*, erythromycin-resistant *Streptococcus pyogenes* and methicillin-resistant or vancomycin-resistant *Staphylococcus aureus* and the concomitant increase in the number of infections [3,6,7,8]. However, little is known about the effect of these particles on eukaryotic microorganisms such as fungi or parasites such as amoebas and, despite the strong antibacterial effect of AgNPs that has been widely described, the mechanisms of this action have not yet been completely elucidated. It has been observed that the use of AgNPs conjugated with multipurpose solutions for contact lenses significantly reduces the adherence of *Acanthamoeba* trophozoites to the surface of the contact lens, decreasing the risk of *Acanthamoeba* keratitis infection. It has also been seen that the conjugation of AgNPs with amphotericin B, means that nystatin has amoebicidal activity, being more effective against *Acanthamoeba castellanii* compared to drugs alone [9,10]. In relation to the size of AgNPs, it is known that the smaller the size, the higher the surface-to-volume ratio and the greater the effectiveness of their use as anti-infective agents. Small-sized AgNPs can interact with the surface of microorganisms altering numerous basic functions such as membrane permeability and respiration [11]. What is more, the silver ions released can interact with the negative charges of the membrane, increasing the toxic effect, and they can also interact with the sulfate or phosphate groups of DNA and proteins in the cytoplasm, causing irreversible damage and inhibiting their growth and replication [1,11]. Moreover, the production of reactive oxygen species (ROS), DNA fragmentation and apoptosis have also been reported [12]. On the other hand, there is evidence that AgNP activity depends not only on their size, but also on their concentration and shape [1], although little is known about the mechanism involved [12,13,14,15,16,17]. It is generally agreed that concentration seems to be key to their cytotoxic action, albeit being dependent on the cell type with which they interact; in the case of bacteria, the greater sensitivity of Gram-negatives to the action of AgNPs was observed [16]. In the case of fungi of the genus *Candida*, the correlation between the fungicidal effect and AgNP concentrations varies depending on the species [15,18].

In addition to this, the shape of the AgNP itself plays an important role and seems to influence its activity [14,15,16,17]. Using different synthesis methods, it is possible to create AgNPs that are spherical, rod-like or triangular in shape, and the latter demonstrate the greatest bactericidal and antifungal potential with respect to dental implants, this apparently being due to their larger active surface area [19]. A similar effect was observed in *Escherichia coli*, where triangular nanoparticles were found to be qualitatively more effective than those that were rod-like or spherical in shape [14]. A new ring-shaped AgNP morphology was recently described, and these NPs demonstrated greater effectiveness against a broad spectrum of bacteria such as *Enterococcus faecalis*, *Streptococcus pneumoniae*, *E. coli* and *Neisseria gonhorreae* in terms of biocidal activity when compared with spherical and wire-shaped NPs [20]. Since the 3 morphologies were obtained by the same procedure, the observed effect highlighted the influence of the shape on the antibacterial activity of AgNPs [20].

On the other hand, the formation of biofilms by a large number of bacteria and fungi is also a serious health problem due to their resistance to existing treatments. Numerous studies analyzed the ability of AgNPs to eradicate these structures, showing that their effectiveness depends on their shape, concentration, size and the species involved in the formation of said structures [1,21,22], although the mechanism underlying their action remains unclear, as in the planktonic cases.

Currently, the increased prevalence of fungal infections, the low availability of antifungal drugs that have low levels of side effects on host cells, and the increase in fungal drug resistance resulted in AgNPs being targeted as potential antifungal agents. AgNPs exhibited very good antifungal activity against *Candida* spp. and *Candida* biofilms, which are implicated in many infections, although their impact on other fungal pathogens is scarcely explored [23,24,25,26]. However, in the case of *Candida*, it was described that AgNPs damage the structure of the cell membrane, producing holes on their surface, increasing membrane permeability and the release of potassium ions, as well as inhibiting cellular processes that are involved in yeast budding through the disruption of the membrane integrity and producing reactive oxygen species that ultimately cause cell apoptosis [18,27]. On the other hand, infections by the free living amoeba *Acanthamoeba* sp. are one of the most difficult to eradicate due to the existence of two different cell forms in the environment, the trophozoite and the cyst. A lack of effective therapeutic agents led to the analysis of the use of NPs conjugated with available drugs for the treatment of this protist, resulting in the significant inhibition of trophozoite growth, along with the inhibition of the encystation and excystation processes [28,29,30].

Taking into account the currently limited knowledge of AgNPs in eukaryotic organisms and that the new ring-shaped NPs known as nanorings (AgNRs) have only been tested in bacteria, the aim of this article was to analyze and compare the antifungal and antiamoebic activity of three different AgNPs, namely nanowires (AgNW), nanospheres (AgNS), and the new AgNRs. All Nps were obtained through the same synthesis process and the tests were carried out using six different fungal species and *A. castellanii*.

## 2. Results

### 2.1. Antifungal Effect of AgNPs Depends on Their Structure and the Species with Which They Interact

**Figure 1:**
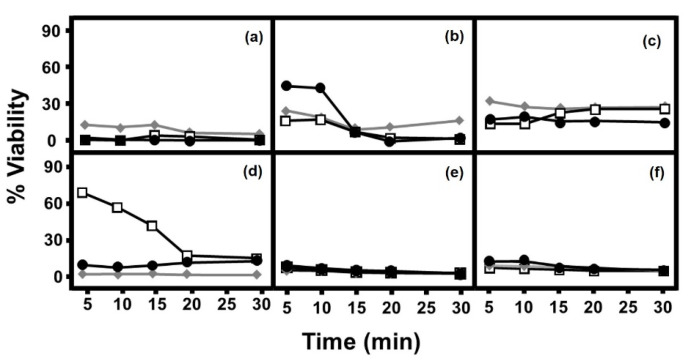
Antifungal effect of AgNPs. (**a**) *C. albicans,* (**b**) *C. parapsilosis*, (**c**) *C. glabrata*, (**d**) *S. cerevisiae*, (**e**) *F. solani*, (**f**) *S. angiospermum*. (◆) AgNSs, (□) AgNWs, (●) AgNRs.

The addition of AgNPs to the different species of fungi produced, in all cases, a biocidal effect although its extent was dependent on the microorganism and the type of AgNP used. (Figure 1). *Candida albicans* and *Candida glabrata* showed high inhibition values, of above 70%, from very short incubation periods, with no notable changes afterwards, and without great differences between the types of nanostructure used (Figure 1a,c). In the case of filamentous fungi *Fusarium solani* and *Scedosporium apiospermum*, a similar result was obtained (Figure 1e,f). In contrast, *Candida parapsilosis* showed differences in inhibition patterns, which were dependent on incubation time, particularly when AgNRs were used (Figure 1b), and *S. cerevisiae* treated with AgNWs also showed an effect clearly dependent on the incubation time (Figure 1d). With both these fungi, the inhibition observed in periods longer than 15 min of incubation were high, and similar for all three nanostructures used.

### 2.2. Fungal Growth Is Influenced by the Presence of AgNPs and by Their Morphology

**Figure 2:**
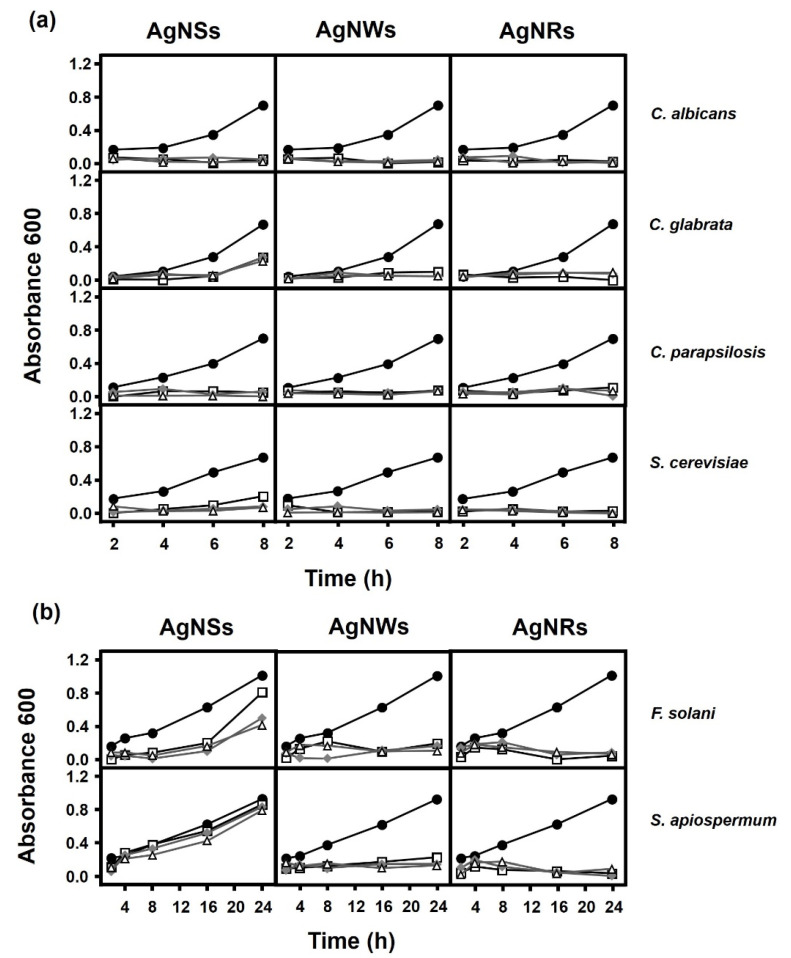
Influence of AgNPs on the growth of fungi species. (**a**) Ratio of yeast:AgNPs used: (●) 1:0, (□) 1:1, (◆) 1:2, (Δ) 1:3. (**b**) Ratio of filamentous fungi:AgNPs used: (●) 1:0, (□) 1:1, (◆) 1:2, (Δ) 1:3.

The effect of AgNPs on growth curves was also analyzed. The results showed that both AgNWs and AgNRs were able to completely inhibit growth in both yeast and filamentous fungi (Figure 2). In contrast, AgNSs showed a variable effect depending on the fungal species involved, that is, they were able to completely inhibit the growth of *C. albicans*, *C. parapsilosis* and *S. cerevisiae* but in the case of *C. glabrata* and *F. solani*, limited growth continued over an extended period, although this was greater in *F. solani* (Figure 2). AgNSs hardly produced any inhibition in the presence of *S. apiospermum* (Figure 2b).

### 2.3. Culture Medium Conditioned by the Presence of AgNPs Shows an Antifungal Effect, although the Effect Is Not Dependent on the Release of Ag Ions

The observed antifungal effect could be due to the direct physical contact of the AgNPs with the microorganisms, or to the toxic conditioning of the culture medium, or both. Taking into account that any such toxic conditioning should be dependent on contact time, both the Saboureaud medium and the deionized water were conditioned with AgNPs using different contact times, which was then followed, after the elimination of the nanoparticles, by immediate contact with *C. albicans*.

**Figure 3:**
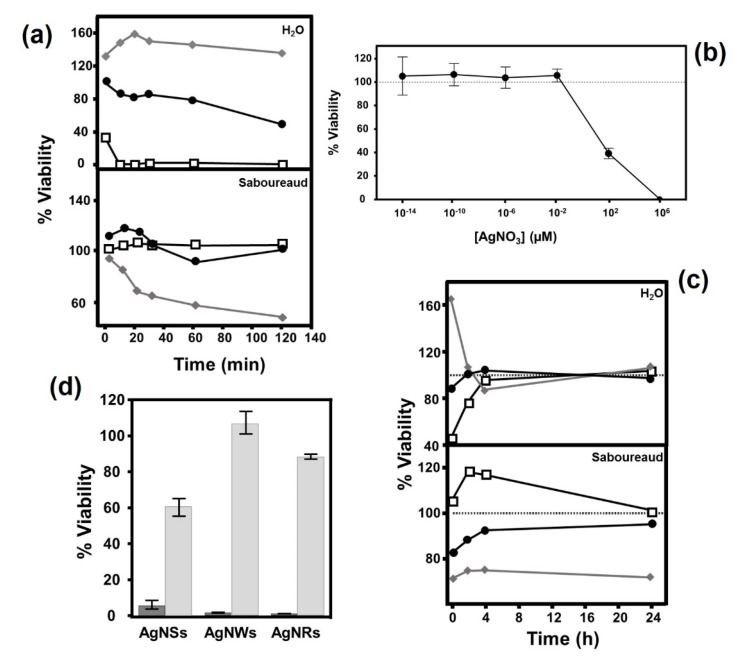
Antifungal effect of medium conditioned by contact with AgNPs. (**a**) Influence of contact time with AgNPs on the antifungal activity of deionized water and Saboureaud conditioned with (◆) AgNSs,(□) AgNWs, (●) AgNRs. (**b**) Effect of AgNO_3_ concentration on the viability of *C. albicans***.** (**c**) Temporal decay of the antibacterial activity of deionized water and Saboureaud from the moment conditioning with (◆) AgNSs, (□) AgNWs or (●) AgNRs was stopped. (**d**) Comparative antifungal effect of AgNPs (black bars) and Saboureaud conditioned (gray bars).

The results showed a variable effect that was highly dependent on whether conditioning was performed with deionized water or culture medium, as well as on the type of AgNP used and on the conditioning time of the medium (Figure 3a). In deionized water, AgNWs induced an intense antifungal effect after only 10 min of incubation, while AgNRs had a smaller effect that increased over time, reaching values close to 60% toxicity after 120 min of incubation. Interestingly, AgNSs had no effect, and an initial increase in the growth of the microorganism was observed (Figure 3a).

In contrast to the above, when conditioning was carried out using Saboureaud medium, AgNWs did not have any effect, AgNRs reduced viability to values below 20% in incubations of 60 min or longer, while AgNSs produced progressive toxic effects that reached more than 70% after 2 h of conditioning (Figure 3a).

To analyze the possibility that the release of Ag ions by the nanostructures could be responsible for the observed antifungal effect, the susceptibility of *C. albicans* to these ions was analyzed by determining its viability after keeping the fungus in a wide range of AgNO_3_ concentrations for 30 min. The results showed that viability was reduced by 50% under these conditions at concentrations of around 10 µM (Figure 3b). Next, the release levels of Ag ions in conditioned medium without AgNPs were determined by ICP-AES, using various combinations of Saboureaud and deionized water with the different nanostructures, both in the presence and absence of *C. albicans*. The results obtained showed that in all cases the concentration of Ag was below 5 ppb (Table 1), which rules out the possibility of attributing the observed antifungal effect to the presence of a sufficient concentration of Ag ions in the conditioned medium.

Having established the existence of the antifungal capacity of the medium conditioned by AgNPs in certain circumstances, the analysis of its duration and stability seemed appropriate. For this, the toxic effect on *C. albicans* cells in Saboureaud medium and deionized water previously conditioned with AgNPs for 1 h, and subsequently maintained for a variable interval after elimination of the nanostructures, was measured. In the case of deionized water, the observed effects dissipated in all cases in less than 4 h. Interestingly, the analysis of the conditioned Saboureaud medium showed that the effects observed with AgNSs, notably higher than those of the other nanostructures, only partially decreased in the first few hours, after which a decrease in viability of around 30% was maintained (Figure 3c).

The existence of antifungal activity in the conditioned medium made it interesting to compare it with the effect observed in the presence of AgNPs. To determine this, Saboureaud was conditioned for 30 min with the different AgNPs, and its effect on the viability of *C. albicans* was compared with yeast incubations in the presence of each AgNP, with incubation for a duration of 30 min in both situations. The results showed that in all cases the reduction in viability was clearly higher when there was physical contact with the AgNPs (>95% for AgNSs, 99.5% for AgNWs and 100% AgNRs), while the medium conditioned by AgNSs reduced viability by 41%, and by 14% when conditioned with AgNRs, while AgNWs had no effect (Figure 3d).

### 2.4. AgNWs and AgNRs Reduce the Proliferation of Acanthamoeba castellanii Trohozoites

**Figure 4:**
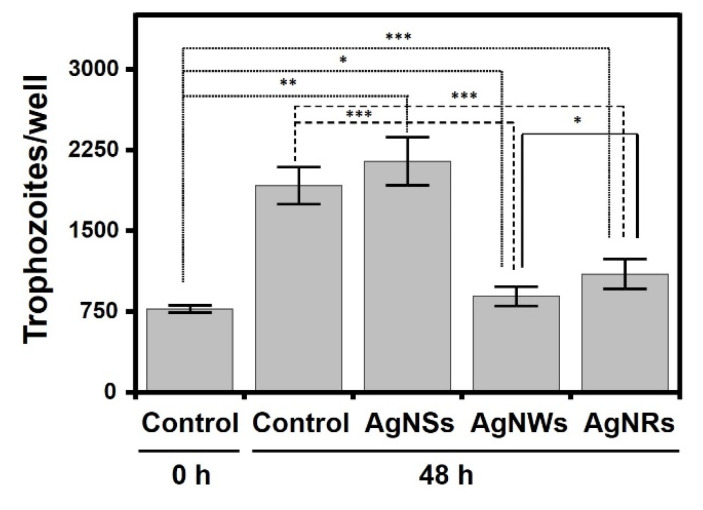
Influence of AgNPs on *A. castellanii* trophozoite proliferation. Statistically significant differences are denoted by ***, ** and * which indicate, respectively, *p* < 0.001, *p* < 0.01 and *p* < 0.05.

*Acanthamoeba* spp. comprises free-living amoeba that can act as opportunistic pathogens. It constitutes a model of a non-fungal eukaryotic microorganisms, which makes it interesting to analyze the effect of AgNPs on its viability. As controls for the experiment, the trophozoites initially present (t = 0 h) and the additional trophozoites formed after 48 h+ of incubation in the absence of AgNPs were used. In all cases, there was some statistically significant proliferation, even in the presence of AgNPs, especially AgNSs (around 195%), although also, to a lesser extent, in the presence of AgNRs (47%) and AgnWs (23%). However, when the proliferation values were compared with those obtained in the absence of AgNPs no significant differences were observed for AgNSs, while AgNRs and AgNWs inhibited proliferation by around 71% and 86%, respectively (Figure 4).

### 2.5. AgNWs Inhibits Germination of A. castellanii Cysts

**Figure 5:**
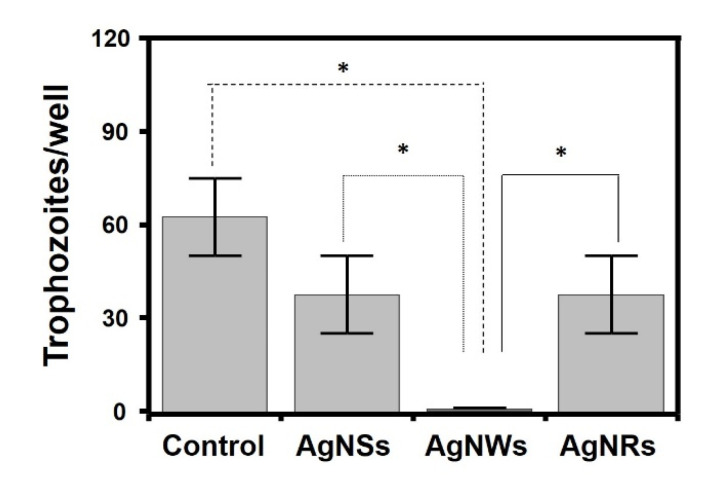
Influence of AgNPs on *A. castellanii* cyst germination. Statistically significant differences are denoted by * which indicate *p* < 0.05.

The effect of AgNPs on the germination of *A. castellanii* cysts was studied by quantifying the appearance of trophozoites from these forms of resistance in the absence and the presence of nanoparticles. In all cases, it was found that AgNPs significantly inhibited germination, although the magnitude of the effect was highly dependent on AgNP morphology. Both AgNSs and AgNRs reduced trophozoite formation by around 40%, while AgNWs were far more effective, completely inhibiting their formation (Figure 5).

## 3. Discussion

The great cytotoxic potential of AgNPs has been widely described in the literature. Numerous studies analyzed their antibacterial capacity against a wide spectrum of microorganisms among which are some such as *S. aureus*, *Staphylococcus epidermidis*, *Bacillus subtilis*, *Klebsiella pneumoniae*, *E. coli*, *Salmonella typhi* [1,17]. However, the antifungal effects of AgNPs received only marginal attention and only a few studies have been published on this area, with most of them taking the genus *Candida*, and specifically *C. albicans*, as a model [23]. Even less is known about their activity against other parasitic eukaryotic microorganisms such as amoeba [31]. The shape, size and concentration of AgNPs are important aspects that influence the effects observed [15,32]. This work analyzed the biocidal capacity of a recently described morphology of AgNPs, namely AgNRs, which have a filament diameter of 80 nm and a ring diameter of between 12 and 18 µm. In addition, the effect of AgNRs was compared against two other types of AgNPs, namely AgNSs and AgNWs. The biocidal effects of AgNSs and AgNWs were described previously in various studies, unlike those of AgNRs, whose activity has only been described in a single study against a broad spectrum of bacteria such as *S. aureus*, *S. epidermidis*, *Streptococcus pyogenes*, *Streptococcus pneumoniae*, *E. faecalis*, *B. globisporus*, *E. coli*, *Serratia marcescens*, *Haemophilus influenzae*, *Klebsiella pneumoniae*, *Neisseria gonorrhoeae* and *Pseudomonas aeruginosa* [20]. Due to the fact that the synthesis of these particles can be carried out by a variety of physical and chemical processes, and that their properties may vary, the comparison of these three morphologies was carried out after obtaining them in parallel using the same methodology.

When the toxic effect of the different AgNPs on fungi was analyzed, a wide range of efficiency could be observed in most cases from the outset and without great differences being observed depending on the nanostructure employed, with the exception of *S. cerevisiae* and *C. parapsilosis*, where AgNWs and AgNRs showed a lower effect at the start of incubation but then achieved inhibition values similar to the other two nanoparticles over longer periods of time. In the case of filamentous fungi, the toxicity values reached were very high, were similar for all three types of AgNPs and did not show great differences with the results obtained in most yeasts. This differs from what has previously been described with bacteria, where differences between the bacteria analyzed were observed and, furthermore, the antibacterial effect of each AgNP varied depending on the microorganism tested and their gram nature [20]. Interestingly, in many cases this inhibitory effect was not as high as in fungi. In addition, AgNSs showed a greater antibacterial effect compared to AgNWs and AgNRs, corroborating the finding that the smaller the size of a nanoparticle, the greater its effectiveness [16,17]. These results, however, differ from what was obtained in this work, where the differences in the size and morphology of the nanoparticles did not seem to show uniform behavior in terms of the level of inhibition when they were in contact with the fungi for short periods of time.

Although the inhibition values obtained over short periods were greater than 70%, the growth of the small surviving fraction was analyzed in longer incubation periods. In this scenario, the efficiency of the AgNPs was different from that observed in the shorter periods. AgNRs and AgNWs displayed a strong inhibitory effect with all fungi analyzed although AgNSs reduced the rate of the fungus growth in a variable manner depending on the fungal species. AgNSs were able to completely inhibit the growth of all fungi with the exceptions of *C. glabrata* and *F. solani*, where limited growth appeared over longer periods, and *S. apiospermum*, where no effect was observed. The effect observed with AgNRs is similar to that obtained in the earlier experiment with bacteria, where they represented the nanoparticles with the greatest inhibitory effect regardless of the gram nature of the bacteria, unlike AgNSs, whose efficacy was lower than the other AgNPs with both bacteria and fungi, although the extent of their impact depended on the microorganism involved [20]. These data do not support the idea that the reduced size of the AgNSs favors their effect in long incubation periods [33], although they do reinforce the notion that the shape of the particle has an important role in terms of its effectiveness. In addition to the physical-chemical characteristics of AgNPs, a key factor in their toxicity is the physiological characteristics of the microorganism on which they act. Thus, the differential response to the action of AgNPs may be due in part to differences in the composition of the cell wall, metabolism or virulence, as occurs in other antimicrobial drug treatments [34]. AgNPs adhere to the cell wall or membrane in both bacteria and fungi, and can penetrate into the cells, leading to serious and varied alterations in cell physiology, causing changes to signal transduction pathways, the induction of oxidative stress and damage to the intracellular structure [16]. The effect of AgNPs on the genus *Candida* has been the subject of some studies and seems to lie in the accumulation of AgNPs on the outside of the fungal wall and the release of Ag ions. As a consequence, alterations in the physiological state of cells and the fluidity of their membranes occur, along with a reduction in the levels of ergosterol and fatty acids in them, inducing cell death [23,35]. This could also explain the differences observed in the viability of the fungal species tested here. On the other hand, some mechanisms do not always involve direct contact with the microorganism and it may even be that the release of Ag ions by the AgNPs is also involved in toxicity [16]. In the case of bacteria, it was described that a large effect occurs when a medium previously conditioned with AgNPs is used, although mortality rates are lower compared to when nanoparticles are present. In the present study, unlike with the bacteria, where a toxic effect was observed in both media and with all types of AgNPs, in *C. albicans* strong differences were observed between type of conditioned medium, deionized water or Saboureaud medium, as well as on the basis of the type of nanoparticle used. While AgNWs produced the highest mortality rate when deionized water was used, in the Saboureaud medium the most effective AgNPs were AgNSs. For their part, AgNRs produced their greatest effect in deionized water. The toxicity of Ag to fungi has been previously described [35] and the possibility that AgNPs release Ag ions has therefore been investigated, finding that the concentration of silver necessary to reduce the viability of *C. albicans* to 50% was 10 μM, a concentration 10 times higher than that required with bacteria [20]. However, the effect does not seem to be due to the release of Ag ions by AgNPs since concentrations of ions sufficient to account for the effect were not detected in either medium. These data suggest the formation of chemical species capable of producing, at least in part, a toxic effect on fungi that is highly dependent on the type of AgNP involved. On the other hand, the average duration of this effect depended on the type of medium and AgNP used. In deionized water, the toxic capacity of all AgNPs progressively dissipated after the first 5 min, similar to what was observed in bacteria. However, analysis of the conditioned Saboureaud medium showed that, in the case of AgNSs, the loss of toxicity over time was limited, unlike that observed in bacteria, where the antibacterial activity in BHI was lost more quickly. This could indicate that either the toxic reactive species released differ depending on the composition of the conditioned medium, or that the effect on fungi is different to in bacteria. Although an antifungal effect was observed in the conditioned medium, it is less than that observed after direct contact with AgNPs, which suggests that, although the mechanism of action is mixed, the AgNP–fungus interaction plays the most important role.

Another eukaryotic opportunistic pathogen is the genus *Acanthamoeba*. Due to the wide distribution of these amoebas in the environment and the variety of diseases that they can cause in humans, together with the difficulties in diagnosing them and the ineffectiveness of treatment, which is often toxic to human cells [28], analyzing the effect of AgNPs on their viability is of great value. To date, few studies have investigated the activity of AgNPs against *A. castellanii*. Cobalt NPs have been studied for their anti-amoebic potential, hexagonal microflakes showing better anti-*Acanthamoeba* results compared to nanoflakes and granular cobalt NPs [36]. In the case of AgNPs, it was observed that their use, alone or conjugated with different drugs, has anti-amoebic and amoebistatic effects, along with inhibited encystation and excystation; in the case of conjugation, this also improves their bioavailability, sustained release, intracellular permeability and efficacy against the eradication of the infection [9,37]. However, there are no data in the literature about the influence of AgNPs morphology on *Acanthamoeba* eradication. The present work was able to establish that the presence of AgNPs reduces the proliferation of trophozoites, albeit without achieving their complete inhibition, when compared to control conditions where AgNPs were not present. The results demonstrate that AgNSs did not have a significant effect, unlike AgNWs, which were the most effective, and AgNRs, which inhibited proliferation, though to a lesser degree. In addition, using AgNPs in the presence of *A. castellanii* cysts lowered the percentage of excystation and, consequently, germination was also notably reduced; therefore, AgNWs once again were identified as the most effective followed by AgNRs and AgNSs. Once again, these results are not consistent with previously described observations that a smaller AgNP size produces a greater effect, instead suggesting the existence of other important factors that influence the mechanism of action of these nanostructures. However, in this work, it seems to be that the shape of an AgNP has a strong influence on its activity against both trophozoites and cyst forms, in contrast to what was observed by other authors, who reported that the shape of an AgNP does not seem to be such an important factor in terms of its activity [38].

## 4. Material and Methods

### 4.1. Fungal Species, Amoeba and Culture Conditions

The fungal species used in this study were *C. albicans*, *C. parapsilosis*, *Candida glabrata*, *S. cerevisiae*, *F. solani* and *S. apiospermum*, with all of them being clinical isolates obtained from the Hospital Universitario Central de Asturias and identified at the species level by MALDI-TOF MS spectrometry (Bruker Daltonics, Bremen, Germany). All species were grown in Saboureaud medium at 37 °C for 48 h. In the case of *F. solani* and *S. angiospermum*, both of which are filamentous fungi, they were grown in a volumetric flask with glass balls at 30 °C for 5 days in a shaking incubator. The amoeba used was *A. castellanii*, in both its active form (throphozoite) and its dormant form (cyst), obtained from Fundación de Investigación Oftalmológica. *A. castellanii* was grown at 30 °C in Peptone yeast glucose medium (PYG) supplemented with 0.5 mM CaCl_2_, 4 mM MgSO_4_, 2.5 mM Na_2_HP_4_, 2.5 mM KH_2_PO_4_, 3.4 mM C_6_H_9_Na_3_O_9_, 0.05 M Fe (NH_4_)_2_(SO_4_)_2_ and penicillin G/streptomycin (5000 IU/mL, 5000 µg/mL).

### 4.2. Synthesis of AgNPs

The synthesis of AgNPs was carried out in the same way as described in Gonzalez-Fernandez et al. [20]. Briefly, this involves the reduction of AgNO_3_ in ethylene glycol in the presence of polyvinylpyrrolidone 360 k. The solution obtained had a transparent to a pearly appearance, denoting the presence of silver nanostructures. The reaction was performed at 170 °C under magnetic stirring. When the reaction was complete, the solution was submerged in ice water until room temperature was reached. After this, the separation and purification steps were carried out. AgNPs were separated by centrifugation and the remaining solution was left to decant for 3 days, after which, from the supernatant obtained, a concentrated solution of AgNRs together with a small quantity of AgNWs was obtained. Finally, the majority of AgNWs were then obtained from the remaining solution. The AgNPs were suspended in water and in order to verify the initial concentrations a gravimetric method was used, the final data being as follows: 1.7 × 10^7^ AgNSs/μL suspension; 6.0 × 10^4^ AgNRs/μL suspension; 2.4 × 10^4^ AgNWs/μL suspension. The structural features of the nanostructures obtained through this process were analyzed by FEG-SEM, and were previously published by our group [20]. These features are AgNSs, 40–60 nm in diameter; AgNRs, 80 nm wire diameter, 12–18 μm (average 14 μm) ring diameter; AgNWs, 200 nm in diameter and 50–100 µm in length. The chemical nature and purity of the silver nanostructures developed were verified by means of an FRX probe, coupled to the FEG-SEM equipment, resulting in a content of 99.9% silver.

### 4.3. Effect of AgNPs on Cell Viability in Fungal Cultures

To test the influence of different AgNPs on yeast and filamentous fungi viability, fungal cultures were grown at an A_600_ of 0.5 and kept at room temperature under agitation with the AgNPs for periods of between 5 and 30 min. The yeast:NP and filamentous fungi:NP ratio were 1:1 and 1:100, respectively. Next, the NPs were removed by centrifugation at 800 rpm for 2 min, and different dilutions of the supernatant were seeded on solid Saboureaud medium plates. After incubation of the plates at 37 °C overnight, the colonies obtained were quantified.

### 4.4. Effect of AgNPs on Cell Growth in Fungal Cultures

The influence of different AgNPs on yeast and filamentous fungi growth was analyzed by incubating the AgNPs with fungal cultures at an A_600_ of 0.02, and using fungi:NP proportions of 1:1, 1:2 and 1:3. The effect on yeast growth was quantified by absorbance after incubation periods of 2, 4, 6 and 8 h. In the case of filamentous fungi, absorbance was determined after incubation for 2, 4, 8, 16 and 24 h.

### 4.5. Determination of the Concentration of Silver Ions Released by AgNPs

Different samples of *C. albicans* in Saboureaud at an A600 of 0.5, sterile Saboureaud, suspensions of *C. albicans* in deionized water at an A_600_ of 0.5 or sterile deionized water were individually treated with an amount of different AgNPs equivalent to a yeast:NP ratio of 1:1 in all cases. After incubating for 30 min with stirring, the suspensions were centrifuged at 3000 rpm for 5 min to discard the AgNPs. The concentration of Ag in the particle-free supernatants was determined by inductively coupled plasma atomic emission spectroscopy (ICP-OES) using an Agilent 5110 ICP-OES Instrument (Agilent, Santa Clara, CA, USA).

### 4.6. Study of the Toxic Effect of Media Conditioned by AgNPs

To analyze the effect that conditioning through the presence of AgNPs might have on fungal viability, aliquots of deionized water or BHI were kept in contact with NPs at different concentrations (10^10^, 2 × 10^10^, 4 × 10^10^, 8 × 10^10^, 16 × 10^10^ Units/L) for a variable time (5, 10, 20, 30, 60 or 120 min, depending on the experiment) with stirring. Next, the AgNPs were removed by centrifugation at 3000 rpm for 5 min, and *C. albicans* was incubated in the conditioned media at an A_600_ of 0.5 for 30 min with stirring. The quantity of nanostructures used in the experiments was adjusted to a fungi:NP ratio of 1:1, equivalent to that used in the cell viability experiments in planktonic fungal cultures. Finally, different dilutions of these cultures were seeded onto solid Saboureaud plates and, after incubation overnight at 37 °C, the colonies obtained were quantified. In order to establish the temporary durability of the culture medium conditioning, aliquots of deionized water and BHI were kept in contact with AgNPs at the same concentrations described in the previous paragraph, after which the AgNPs were removed by centrifugation at 3000 rpm for 5 min, and the supernatant was kept at room temperature for 0, 2, 4 and 24 h. Each sample of conditioned medium was incubated with *C. albicans* at an A_600_ of 0.5 for 30 min with stirring, and the quantification was carried out by dilution in solid BHI medium as described above.

### 4.7. Effect of AgNPs on Cell Viability in Trophozoites and Cyst Form of A. castellanii

The influence of NPs on the trophozoite and cyst forms of *A. castellanii* was analyzed by culturing the amoeba in 24-well plates. The trophozoites were coincubated with the different NPs at 30 °C for two days in the trophozoite:NP 1:250 ratio, while the cysts were coincubated with the NPs at 30 °C for 8 days, using a cyst:NP ratio of 1:500. After this time, the trophozoites were quantified using a Neubauer chamber. The cysts were also analyzed following the same method, although the quantification was carried out after both 4 and 8 days of coincubation.

### 4.8. Statistical Analysis

The results were analyzed using a Kruskal–Wallis test in the Statistics for Windows program (Statsoft Inc.; Tulsa, OK, USA). The differences were considered significant when *p* < 0.05.

## 5. Conclusions

This work analyzed the antifungal and antiamoebic properties of AgNRs, a new type of AgNP that has recently been described, and compared the results with those of two other morphologies, namely AgNSs and AgNWs. AgNRs showed antifungal activity in all cases although the effectiveness varied depending on the fungus and incubation time involved. In addition, they were able to inhibit the growth of yeasts and in the case of filamentous fungi, the AgNRs showed great effectiveness compared to the other AgNPs. In addition, AgNPs were able to partially inhibit the proliferation of trophozoites and the germination of cysts of *A. castellanii*, the best results being seen with AgNWs. These results open the door to new and effective antimicrobial therapies as an alternative to the use of antifungals or antiamoebic drugs, thus avoiding the constant appearance of resistance and addressing the difficulty of eradicating infections.

## Figures and Tables

**Table 1:** Presence of Ag ions in AgNP-free conditioned medium analyzed by inductively coupled plasma atomic emission spectroscopy (ICP-OES).

Sample	[Ag] (ppb)
H_2_O milliQ	<5
H_2_O milliQ + AgNSs	<5
H_2_O milliQ + AgNWs	<5
H_2_O milliQ + AgNRs	<5
H_2_O milliQ + AgNSs + *C. albicans*	<5
H_2_O milliQ + AgNWs + *C. albicans*	<5
H_2_O milliQ + AgNRs + *C. albicans*	<5
Saboureaud	<5
Saboureaud + AgNSs	<5
Saboureaud + AgNWs	<5
Saboureaud + AgNRs	<5
Saboureaud + AgNSs + *C. albicans*	<5
Saboureaud + AgNWs + *C. albicans*	<5
Saboureaud + AgNRs + *C. albicans*	<5

## Data Availability

The data presented in this study are contained within the article.
